# Dynamic Status of REST in the Mouse ESC Pluripotency Network

**DOI:** 10.1371/journal.pone.0043659

**Published:** 2012-08-28

**Authors:** Sanjay K. Singh, Bethany L. Veo, Mohamedi N. Kagalwala, Weiwei Shi, Shoudan Liang, Sadhan Majumder

**Affiliations:** 1 Department of Genetics, The University of Texas M. D. Anderson Cancer Center, Houston, Texas, United States of America; 2 Department of Bioinformatics, The University of Texas M. D. Anderson Cancer Center, Houston, Texas, United States of America; 3 Department of Neuro-Oncology, The University of Texas M. D. Anderson Cancer Center, Houston, Texas, United States of America; 4 The Brain Tumor Center, The University of Texas M. D. Anderson Cancer Center, Houston, Texas, United States of America; 5 Program in Genes and Development, The University of Texas Graduate School of Biomedical Sciences at Houston, Houston, Texas, United States of America; Baylor College of Medicine, United States of America

## Abstract

**Background:**

REST is abundantly expressed in mouse embryonic stem cells (ESCs). Many genome-wide analyses have found REST to be an integral part of the ESC pluripotency network. However, experimental systems have produced contradictory findings: (1) REST is required for the maintenance of ESC pluripotency and loss of REST causes increased expression of differentiation markers, (2) REST is not required for the maintenance of ESC pluripotency and loss of REST does not change expression of differentiation markers, and (3) REST is not required for the maintenance of ESC pluripotency but loss of REST causes decreased expression of differentiation markers. These reports highlight gaps in our knowledge of the ESC network.

**Methods:**

Employing biochemical and genome-wide analyses of various culture conditions and ESC lines, we have attempted to resolve some of the discrepancies in the literature.

**Results:**

We show that Rest+/− and Rest−/− AB-1 mutant ESCs, which did not exhibit a role of REST in ESC pluripotency when cultured in the presence of feeder cells, did show impaired self-renewal when compared with the parental cells under feeder-free culture conditions, but only in early passage cells. In late passage cells, both Rest+/− and Rest−/− AB-1 ESCs restored pluripotency, suggesting a passage and culture condition-dependent response. Genome-wide analysis followed by biochemical validation supported this response and further indicated that the restoration of pluripotency was associated by increased expression of the ESC pluripotency factors. E14Tg2a.4 ESCs with REST-knockdown, which earlier showed a REST-dependent pluripotency when cultured under feeder-free conditions, as well as Rest−/− AB-1 ESCs, showed no REST-dependent pluripotency when cultured in the presence of either feeder cells or laminin, indicating that extracellular matrix components can rescue REST's role in ESC pluripotency.

**Conclusions:**

REST regulates ESC pluripotency in culture condition- and ESC line-dependent fashion and ESC pluripotency needs to be evaluated in a context dependent manner.

## Introduction

Pluripotent mouse embryonic stem cells (mESCs) derived from the inner cell mass of the mammalian blastocyst are capable of forming all the tissues of the organism and possess the ability to self-renew in an undifferentiated manner. Understanding the mechanisms that control the self-renewal and pluripotency of ESCs has far-reaching implications for the fields of developmental biology, regenerative medicine, and oncology [1], [2], [3]. The recent successes with reprogramming somatic cells into induced pluripotent stem-like cells have brought intense enthusiasm to this area of research [4], [5], [6], [7], [8], [9], [10], [11]. These findings suggest that factors such as Oct4, Nanog, and Sox2 are core components of a large, interconnected network that regulates self-renewal and pluripotency in both mouse and human ESCs [2], [12], [13], [14]. This network is likely regulated by maintenance factors that are triggered by cellular and environmental signals [15]; these factors both protect the self-renewal state from spurious signals and cause differentiation of the cell when required.

REST is a transcriptional repressor that was originally discovered to be a repressor of many neuronal differentiation genes [16], [17]. Experiments with Rest mutant mice supported REST's role in neurogenesis [18]. However, later reports indicated that REST can potentially repress about a thousand genes and affect various cellular functions in a context-dependent fashion [19], [20], [21], [22], [23]. Although REST expression is higher in ESCs than in most other cell type [24], its function in the self-renewal network is unclear. REST has been reported to maintain mESC self-renewal and pluripotency by directly suppressing miR-21 [25]. In these studies, loss of REST in ESCs accompanied expression of many differentiation markers, including Gata4. In contrast, others found REST to have no role in the maintenance of mESC pluripotency [26], [27], [28] and loss of REST did not change expression of differentiation markers including Gata4 [27], [28]. Yet, another report found that REST was not required to maintain ESC pluripotency and, in contrast, loss of REST actually caused decreased expression of differentiation markers, including Gata4 [29]. However, genome-wide promoter occupancy assays have shown that REST indeed is an integral part of the ESC pluripotency network [30], [31], [32], [33], [34], raising the interesting question whether such genome-wide assays truly reflect the biology of ESC pluripotency. Thus, these conflicting results have created a gap in our knowledge of the mESC network, particularly with respect to REST. Using wild-type, Rest^+/−^ and Rest^−/−^ ESCs and conditions used by different laboratories, we determined how REST functions in the mESC pluripotency network in coordination with other factors. The work presented here resolves some of the apparent discrepancies in the field.

## Materials and Methods

### Mouse ESC lines and culture conditions

E14Tg2a.4 were purchased from Bay Genomics (MMRRC item #015890-UCD-CELL) and parental AB1 (N6: Wild-type), N9 (Rest^+/−^) and N8 (Rest^−/−^) were obtained as gifts from Amanda Fisher Laboratory [27] (originally obtained in presence of feeder cells and were maintained in presence of feeder layer and LIF; passage numbers in the manuscript represent passage in absence of feeder cells), were cultured without feeder cells in presence of 1000 units ml^−1^ LIF (ESGRO) on gelatin-coated tissue culture dishes [35]. Inactivated (Mitomycin C-treated) mouse embryonic fibroblasts (MEFs) were used as feeder layer in addition to LIF in some experiments. MEFs were obtained from ATCC (SCRC-1040™, ATCC) and were used at passage no more than four passages (P4). Laminin coated plates were made by incubating the plates with 5 µg/ml Laminin in PBS (invitrogen, # 23017-015) at 4°C overnight.

### Western blotting and quantitative RT-PCR

Whole-cell extracts were prepared, and approximately 30 µg of proteins were resolved on SDS-PAGE using antibodies against REST (#07–579, Upstate), Oct4 (#ab19857, Abcam), Sox2 (#ab15830, Abcam, #2748, Cell Signaling), β-actin (A5316, Sigma), α-tubulin (#MMS-407R, Covance), and NANOG (A300–398, Bethyl Laboratories) per the manufacturers' recommendations. Secondary antibodies labeled with IRDye infrared dyes (IRDye 680LT and IRDye 800CW) were used. Western blot quantification were performed using Odyssey 2.1 software from *LI-COR*. Total RNA was extracted using TRIzol (Invitrogen), and approximately 500 ng of total RNA was used as template for cDNA synthesis. For quantitative reverse transcriptase PCR, cDNAs were prepared using RT PCR kit from Thermo Fisher Scientific (AB-4113) followed by real-time PCR using SYBR Green PCR mater mix (4309155, Applied Biosystems) as per manufacturers' recommendations. All qRT-PCRs were performed in triplicates.

The following gene-specific primers were designed: REST forward1, 5′-AGCGAGTACCACTGGAGGAA-3′; REST reverse1, 5′-CTGAATGAGTCCGCATGTGT-3′; REST forward2, 5′- AGGGTTGGCTGGTAAAGTGACTGA-3′ ; REST reverse2, 5′- CAAGTGGCGATTGAGGTGTTTGCT-3′; REST forward3, 5′- TGTGAACGAGGGACCAGTGACAAA-3′; REST reverse3, 5′- CGGCGTCTTCTTTGTGCCTTTCTT-3′. The following primers were based on published sequences: Gapdh, Oct4, Nanog, [36]; Sox2 [25] and lineage specific markers [25].

### shRNA and RNAi-mediated knockdown and self-renewal assay

RNAi-mediated knockdown of REST was carried out per the manufacturer's protocol (Dharmacon). siREST (400 pmole), and nontargeting siRNA (400 pmole) were used in nucleofection using AMAXA. Approximately 1000 cells post-transfection were seeded per well in triplicates of 6-well plates for self-renewal assay. Colonies were assayed for alkaline phosphatase activity 3 days after transfection. shRNA mediated knockdown was performed using 1 MOI of non-targeting control (RHS4372, Thermo scientific) and REST (V3LMM_509606, Thermo scientific) specific lentiviruses. Puromycin selection (1.5 µg/ml) was performed 48 hours post transduction. Puromycin-resistant and GFP positive cells were selected and expanded for several generations for further experiments after FACS analysis. Percentages of self-renewing colonies from three experimental replicates were plotted. Standard error of means was calculated and is depicted as error bars in the figures.

### Microarray and bioinformatic analysis

One sample from each cell line (N6 (WT), N9 (Rest^+/−^), and N8 (Rest^−/−^)) cultured in parallel for passage 2 and passage 10 (after removal of feeder cells) were used for microarray analysis. Total RNA was isolated using Trizol method from N6 (WT), N9 (Rest^+/−^), and N8 (Rest^−/−^) cells grown for various passages. Cleanup was performed for all samples using Qiagen RNeasy mini kit. Sample RNA concentration was determined with a Nanodrop ND-1000 Spectrophotometer and RNA quality was verified with an Agilent 2100 Bioanalyzer using a RNA Nano Chip. RNA samples displaying no visible degradation in the Bioanalyzer analysis with two sharp ribosomal peaks were deemed acceptable for further processing. Affymetrix's GeneChip IVT Express kit was used for cDNA synthesis and *in vitro* transcription. Affymetrix GeneChip Mouse Genome 430 2.0 was used in this study. A minimum of greater than or equal to 2.0 fold change was established as the criteria during analyses. All data was normalized using the RMA expression statistical analysis (Robust Multi-array Analysis. Venn Diagram between N6 (WT), N9 (Rest^+/−^), and N8 (Rest^−/−^) was plotted after calculating the fold changes for genes from passage 2 to 10 for each cell types (N6, N9 and N8) and selected those with their absolute fold changes more than two as differential expressed ones. For overlap and its significance with FunGenES database, we selected 50 most up-regulated genes and 50 most down-regulated genes in N9 (Rest^+/−^) and N8 (Rest^−/−^) cells during the transition from passage 2 to passage 10 and compared them with FunGenES database: http://biit.cs.ut.ee/fungenes/ for their overlap (concordance) in the regulation direction. The statistical significance (p-value) of the overlap was further evaluated using hypergeometric distribution.

### Ingenuity Pathway Analysis (IPA)

The dataset obtained from microarray analysis of samples from passages 2 and 10 for each cell line was uploaded to IPA server. The genes with more than or equal to 2 fold change in expression were included for analysis. For all the IPA analyses, the significance value associated with Functional Analysis for the dataset is a measure of the likelihood that the association between a set of Functional Analysis genes in experimental samples and a given process or pathway is due to random chance. The smaller the p-value the less likely that the association is random and the more significant the association. In general, p-values less than 0.05 indicate a statistically significant, non-random association. The p-value associated with a biological process or pathway annotation is a measure of its statistical significance with respect to the Functions/Pathways/Lists Eligible molecules for the dataset and a Reference Set of molecules (which define the molecules that could possibly have been Functions/Pathways/Lists Eligible). The p-value is calculated with the right-tailed Fisher's Exact Test.

## Results

### Rest^+/−^ and Rest^−/−^ AB-1 ESCs cultured in the absence of feeder cells show impaired self-renewal that is restored upon prolonged culture

Previously, using two Rest^+/−^ mutant and their parental E14Tg2a.4 mESC cell lines cultured in the absence of mouse embryonic fibroblast feeder cells, we found that REST maintains the self-renewal and pluripotency of ESCs [25]. We cultured these cells without feeder cells because we wanted to use a system in which the ESC pluripotency pathways are not influenced by the feeder cells. In addition, we chose the E14Tg2a.4 mESC line because these cells were generated to grow without feeder cells. However, using Rest mutant (N9 (Rest^+/−^) and N8 (Rest^−/−^)) and their parental AB-1 N6 (WT) cell lines cultured in the presence of feeder cells, others found that REST does not have a role in the maintenance of mESC self-renewal and pluripotency [27]. To gain insight into this discrepancy, we obtained these cell lines [N6 (WT), N9 (Rest^+/−^) and N8 (Rest^−/−^)] from the investigators and maintained them in the presence of feeder cells plus LIF. We then took aliquots of the N6 (WT), N9 (Rest^+/−^) and N8 (Rest^−/−^) cells, cultured them with LIF, but in the absence of feeder cells, and measured their self-renewal capacity using alkaline-phosphatase (AP) assays.

**Figure 1:**
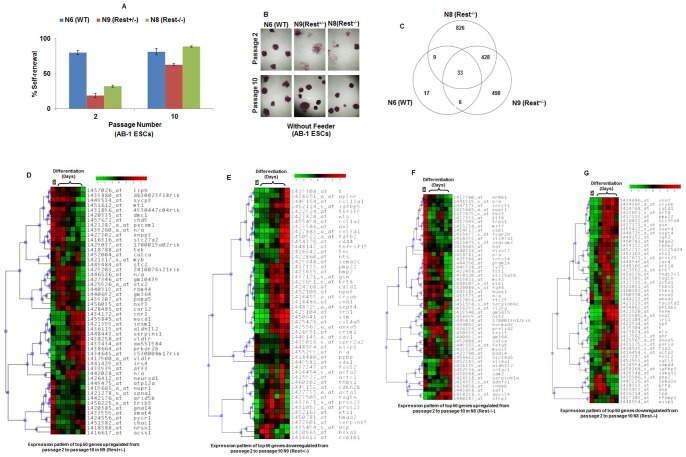
Rest+/− and Rest−/− AB-1 ESCs cultured in the absence of feeder cells show impaired self-renewal that is restored upon prolonged culture. (A, B) Alkaline phosphatase based self-renewal assay shows that the self-renewal efficiency of the N9 (Rest^+/−^) and N8 (Rest^−/−^) ESCs was significantly lower than that of the parental AB-1 ESCs at passage 2 but not at passage 10. (A) quantification of self-renewal assays. (B) representative pictures of colonies from self-renewal assays. The numbers under the lines show p-values (n = 3). Error bars are standard error of means. Cells were cultured in absence of feeder cells and in presence of LIF. (C) Venn diagram showing comparison of genes that showed a significant change in expression during the transition between passage 2 and passage 10 by genome-wide expression profiling. N9 (Rest^+/−^) and N8 (Rest^−/−^) cells showed significant changes in gene expression (N9 ∼965 and N8 ∼1296) compared to WT cells (∼65). (D, E, F and G) Heat map showing comparison of 50 genes with the highest fold changes that were either (D and F) up-regulated or (E and G) down-regulated in Rest+/− and Rest−/− cells during the transition from passage 2 to passage 10 to the expression of these genes in ES and differentiating cells based on FunGenES database.

The control N6 (WT) line morphologically showed minimal differentiation, whereas both the mutant N9 (Rest^+/−^) and N8 (Rest^−/−^) AB-1 lines showed extensive differentiation within 2 passages after they were shifted to feeder-free culture conditions (data not shown), similar to what was reported previously with the E14Tg2a.4 cell line and its derivative REST^+/−^ mutant cells [25]. Surprisingly, upon prolonged culture (10 passages), both the mutant cell population morphologically looked similar to the wild-type cells. To analyze this phenomenon further, we performed AP assays. As shown in Figure 1A, the self-renewal efficiencies of both the mutant lines were significantly lower than that of the parental AB-1 ESCs at passage 2 but not at passage 10 (Figure 1A, quantification of self-renewal assays; Figure 1B, representative pictures of colonies from self-renewal assays) (see also Figure S1 for picture of representative dishes and calculations of percent self-renewal). At passage 2, the N9 (Rest^+/−^) and N8 (Rest^−/−^) lines had fewer compact colonies and less self-renewal efficiency compared with the parental line. In contrast, at passage 10, the self-renewal efficiency of the parental line stayed the same (no statistical difference), whereas both the mutant lines recovered their self-renewal capacity with strong statistical significance and were now equivalent to the wild-type line (Figure S1). These results indicated that REST mutant AB-1 ESCs cultured in the absence of feeder cells produced impaired self-renewal during early passage and this deficiency is restored upon prolonged culture. Thus, these results explain the discrepancy seen between the contrasting reports reported earlier [25], [27]. Interestingly, although both Rest^+/−^ and Rest^−/−^ cells show drastic reduction in self-renewal at passage 2 and subsequent restoration at passage 10 when compared with WT cells, Rest^−/−^ cells show higher ability of self-renewal when compared with Rest^+/−^ cells at both passage 2 and passage 10. One of the possibilities to explain this phenomenon is that complete loss of REST protein renders these cells predisposed to faster restoration of self-renewal by a yet-unknown mechanism (see Discussion).

### Genome-wide analysis of Rest+/− and Rest−/− AB-1 ESCs cultured in the absence of feeder cells shows impaired self-renewal that is restored upon prolonged culture

To understand the mechanisms behind the restoration of self-renewal in the mutant cells, we determined the mRNA expression profiles of N6 (WT), N9 (Rest^+/−^), and N8 (Rest^−/−^) cells that were cultured after either passage 2 or passage 10. A comparison of gene expression profiles between N9 (Rest^+/−^), N8 (Rest^−/−^) and N6 (WT) cells cultured for passage 2 and published lineage specific markers (the lists are from microarray profiling studies of lineage specific differentiation of ES cells) [37]. We then used Ingenuity Pathway Analysis (IPA) to overlay N9 (Rest^+/−^), N8 (Rest^−/−^) and N6 (WT) expression profiles with known lineage specific markers). Such analysis revealed that N9 (Rest^+/−^) and N8 (Rest^−/−^) cells showed lower expression of many ESC-specific genes and higher expression of ectoderm-, mesoderm-, and endoderm-specific lineage genes (Table 1). These results indicated that N9 (Rest^+/−^) and N8 (Rest^−/−^) cells were less similar to ESCs than the wild-type cells at passage 2 and supported the earlier AP assays described in Figures 1A and 1B.

**Table 1:** Comparison of gene expression levels for N9 (Rest^+/−^)/N6 WT and N8 (Rest^−/−^)/N6 (WT) cultured on gelatin for passage 2.

	ES cell specific		Ectoderm		Mesoderm		Endoderm	
Samples	Gene Name	Fold Change	Gene Name	Fold Change	Gene Name	Fold Change	Gene Name	Fold Change
N9 (REST+/−)/WT (Passage 2)	AASS	−4.94684	CCND2	6.69444	AMOT	5.502564	AMOT	5.502564
	AF067061	−6.40663	CDH2	10.43632	APLNR	4.581207	ANXA4	2.557478
	ENPP3	−2.08469	CTNND2	3.462363	BMP2	10.40963	APOC2	−2.05542
	FBXO15	−2.57255	CYR61	5.236435	CDH11	2.461676	CDX2	2.924592
	FGF4	−3.64627	DBN1	4.776496	CDH2	10.43632	Ceacam1	−5.06069
	FMR1NB	−17.5118	EFNB2	2.843245	CDX2	2.924592	CITED1	2.527414
	HSD17B14	−2.55802	GLI3	3.036916	CYP26A1	3.256904	CLDN6	2.731065
	HSF2BP	−5.10143	HIPK2	3.117166	FGF5	2.412625	KRT7	3.335138
	KLF2	−2.08667	KBTBD11	−13.088	FGF8	4.674094	LGALS1	3.610094
	MAEL	−2.05708	MARCKS	2.904111	FST	4.833413	NPNT	4.183228
	MYLPF	2.252683	NEDD9	2.666821	FZD2	3.702094	SLC39A8	2.301578
	PLA2G1B	2.20061	NNAT	7.103741	IGFBP5	10.80621	SPINK1	4.92142
	PNMA5	−19.4686	PAX6	−2.25725	NKX1-2	5.025234	TMPRSS2	2.788552
	PRDM14	−2.58181	PIK3R3	3.060537	OTX2	−3.90586		
			PKIA	1.993754	PDZRN3	3.783262		
			PLAGL1	2.296666	PITX2	2.154942		
			RARB	2.414682	PKDCC	3.744951		
			SOX11	4.033373	ROR1	4.044366		
			TOX3	3.176028	RSPO3	2.064006		
			WLS	8.797523	SLC39A8	2.301578		
			ZEB2	4.061111	SP5	6.494405		
					SPRR2A	10.66781		
					T	26.24359		
					TNFRSF19	5.624426		
N8 (REST−/−)/WT (Passage 2)	AASS	−2.49	BCL2	2.11	AMOT	4.61	AMN	2.255
	ESRRB	−2.46	CCND2	4.22	BMP2	2.04	AMOT	4.61
	FGF4	−4.48	CDH2	5.44	CDH2	5.44	ANXA4	2.1
	FMR1NB	−3.26	CYR61	6.08	CDX2	2.72	APOC2	−2.12
	HSD17B14	−2.27	DBN1	3.1	ENPP2	2.44	CACNA1B	4.78
	HSF2BP	−2.42	EFNB2	2.93	F5	2.32	CDX2	2.72
	KLF4	−2.54	GLI3	2.63	FGF5	6.84	Ceacam1	−2.88
	LRRC34	−2.54	HIPK2	2.33	FGF8	5.18	CITED1	4.03
	NR0B1	−2.74	INSM1	2	FST	4.95	CLDN6	3.35
	PNMA5	−2.18	KBTBD11	−7.33	FZD2	3.56	CUBN	2.37
	PRDM14	−2.92	NEDD9	2.41	NKX1-2	2.09	KRT7	3.61
			NNAT	5.53	PITX2	2.88	SLC39A8	5.52
			PKIA	2.16	PKDCC	4.56	SOX17	2.14
			RBMX	−2.04	RBMX	−2.04	SPINK1	2.26
			SCG3	11.79	SLC39A8	5.52	TMPRSS2	2.22
			SCG5	11.02	SOX17	2.14		
			SOX11	2.75	SP5	6.31		
			TOX3	5.87	SPRR2A	4.43		
			WLS	3.24	T	22.59		
					TNFRSF19	3.37		
					TOX3	5.87		
					TRH	2.24		

Lowered expression (−ve values); increased expression (+ values).

To determine what happens to the N9 (Rest^+/−^), N8 (Rest^−/−^) and N6 (WT) cells during the transition between passage 2 and passage 10, we compared their gene expression profiles [37]. Genes that showed a significant change in expression during the transition between passage 2 and passage 10 were scarce in wild-type cells (about 65 genes) but plentiful in N9 (Rest^+/−^) (965 genes) and N8 (Rest^−/−^) (1296 genes) (Figure 1C), which supports the idea that the N9 (Rest^+/−^) and N8 (Rest^−/−^) cells undergo a strong change in global gene expression upon prolonged culture.

We then compared alterations in expression profiles of N9 (Rest^+/−^), N8 (Rest^−/−^) and N6 (WT) cells over passages (from passage 2 to passage 10) (Table 2). The wild-type cells showed minimal alteration in either ESC-, ectoderm-, mesoderm- or endoderm-specific genes during the transition between passage 2 and 10. In contrast, the N9 (Rest^+/−^) and N8 (Rest^−/−^) cells showed significant increase in ESC specific genes and significant decrease in all three lineage-specific genes, again supporting our AP-based evidence that while the wild-type cells represent pluripotent state and remain largely unchanged during the prolonged culture, the impaired self-renewal in Rest mutant cells seen in early passage cells is restored in late passage cells.

**Table 2:** Comparison of expression levels of genes for WT (N6), N9 (Rest^+/−^) and N8 (Rest^−/−^) during the transition between passage 2 and passage 10 when cultured on gelatin.

	ES cell specific		Ectoderm		Mesoderm		Endoderm	
Samples	Gene Name	Fold Change	Gene Name	Fold Change	Gene Name	Fold Change	Gene Name	Fold Change
WT (N6) Passage10/Passage2	-	-	-	-	PITX2	−2.69	KRT7	−2.14
REST^+/−^ (N9) Passage10/Passage2	ENPP3	4.248941	CCND2	−3.58579	AMOT	−4.43156	AMOT	−4.43156
	FMR1NB	2.167808	CDH2	−3.60351	APLNR	−6.06492	CACNA1B	2.026263
	LEFTY1	2.163072	CRABP1	2.569474	BMP2	−6.78678	CDX2	−2.25219
	MYLPF	−2.84712	CTNND2	−4.17178	CDH11	−3.05245	CITED1	−3.61614
	PNMA5	3.830756	CYR61	−2.2737	CDH2	−3.60351	KRT7	−3.92622
	SLC27A2	3.913316	DBN1	−2.04125	CDX2	−2.25219	LGALS1	−3.98939
	SYCP3	2.730131	DLL1	2.556731	CYP26A1	−3.96947	NPNT	−7.91072
			GLI3	−2.09621	EOMES	−2.54611	TMPRSS2	−3.05955
			IGDCC3	−2.00022	FGF8	−5.25247		
			INSM1	2.637314	FST	−2.74678		
			MARCKS	−2.32126	FZD2	−2.19053		
			MEGF10	−2.21195	IGFBP5	−12.4023		
			NEDD9	−2.97277	IRS4	2.871026		
			NNAT	−4.09559	NKX1-2	−4.72778		
			PAX6	2.583957	OTX2	4.781368		
			PIK3R3	−2.84254	PDZRN3	−2.63348		
			PKIA	−2.06279	PKDCC	−2.06611		
			PLAGL1	−2.89334	ROR1	−3.57292		
			SOX11	−2.52691	RSPO3	−2.48247		
			WLS	−10.0147	SP5	−5.23499		
			ZEB2	−3.20816	SPRR2A	−12.2506		
					T	−23.0839		
					TNFRSF19	−8.28107		
REST^−/−^ (N8) Passage10/Passage2	EIF1AX	2.02	CDH2	−2.82	AMOT	−5.49	AMOT	−5.49
	ESRRB	3.05	CRABP1	2.6	CDH2	−2.82	CACNA1B	−3.92
	FGF4	3.46	CTNND2	−2.6	CDX2	−2.3	CDX2	−2.3
	FMR1NB	2.95	CYR61	−4.29	DCN	−2.58	Ceacam1	3.56
	KLF4	4.53	DBN1	−2.3	ENPP2	−4.09	CITED1	−5.89
	LAPTM5	2.37	DPYSL4	3.61	F5	−4.59	CLDN6	−3.66
	LRRC34	5.14	EFNB2	−4.42	FGF5	−4.64	ESRP1	−2.29
	NR0B1	4.51	FJX1	−2.39	FGF8	−5.81	GATA6	−2.11
	PNMA5	2.94	GLI3	−3.54	FST	−2.48	KRT7	−7.25
	PRDM14	5.16	IGDCC3	−2.26	FZD2	−10.06	LGALS1	−2.84
	RNF17	2.46	INSM1	−2.61	HAS2	−2.53	NPNT	−2.92
	SPP1	2.34	KBTBD11	2.47	ITGB8	−2.01	RAB15	−2.2
	SRGN	−2.67	LFNG	−2.27	NKX1-2	−2.31	RIPK4	−2.91
	SYCP3	2.81	MARCKS	−2.42	OTX2	2.28	SLC39A8	−7.84
	TRIML1	2.23	NEDD9	−4.31	PITX2	−5.78	SOX17	−2.36
	ZFP42	2.24	NES	−2.42	PKDCC	−3.72	TMPRSS2	−3.46
			NNAT	−7.26	RBMX	3.08		
			NRARP	−2.27	SLC39A8	−7.84		
			PIK3R3	−2.72	SOX17	−2.36		
			PKIA	−3.13	SP5	−6.91		
			PLAGL1	−4.08	SP8	−2.03		
			PTN	−5.75	SPRR2A	−5.26		
			RBMX	3.08	T	−21.84		
			SOX11	−4.74	TNFRSF19	−4.85		
			ST8SIA1	3.07				
			STXBP6	−2.33				
			TOX3	−4.09				
			WLS	−6.7				

Decreased expression (−ve values); increased expression (+ve values).

We then selected the 50 genes with the highest fold changes that were either up-regulated or down-regulated in N9 (Rest^+/−^) and N8 (Rest^−/−^) cells during the transition from passage 2 to passage 10 (Table S1 (for N9 (Rest^+/−^)) and Table S2 (N8 (Rest^−/−^)) for specific fold-changes in gene expression levels) and compared them to an additional independent database (FunGenES database: http://biit.cs.ut.ee/fungenes/) [38] for their concordancy in up- or down-regulation (Table S1 and Table S2). The wild-type ESC expression profile was not included in this analysis because these cells did not show major changes in their gene expression profile from passage 2 to passage 10. Results indicated that the up-regulated genes corresponded mostly to genes expressed in self-renewing ESCs (the concordancy is 37 out of 50; P = 0.00999 for N9 (Rest^+/−^) and 39 out of 50; P = 0.00163 for N8 (Rest^−/−^)) (Figure 1D and Figure 1F, see Table S3), whereas the down-regulated genes corresponded mostly to genes expressed in differentiating embryoid bodies (EB6) [similarly, the concordancy is 46 out of 50; P = 3.30E-13 for N9 (Rest^+/−^) and 45 out of 50; P = 4.12E-12 for N8 (Rest^−/−^)] (Figure 1E and Figure 1G, see Table S3). Thus, supporting the AP assays seen above, the genome-wide analysis using both IPA and FunGenES databases indicated that, while the wild-type cells showed similarity to pluripotent ESCs and remain unchanged from passage 2 to passage 10, the N9 (Rest^+/−^) and N8 (Rest^−/−^) cells showed more similarity to differentiating cells at passage 2 while they showed more similarity to self-renewing ESCs in passage 10.

### In genome-wide analysis, restoration of the impaired self-renewal and pluripotency of Rest+/− and Rest−/− AB-1 ESCs associates, at least in part, with increased ESC pluripotency pathways

**Figure 2:**
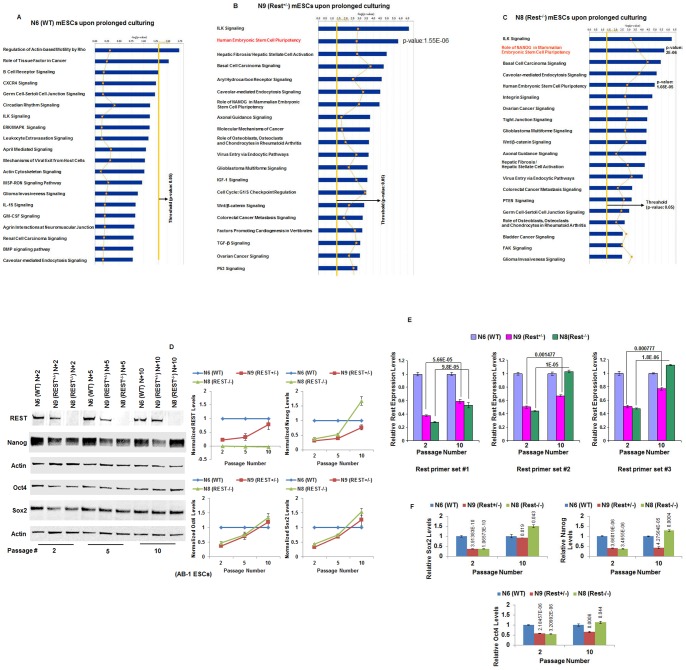
Restoration of the impaired self-renewal and pluripotency of Rest+/− and Rest−/− AB-1 ESCs upon prolonged culture correlates, at least in part, with increased ESC pluripotency pathways. (A, B and C) Ingenuity pathway analysis comparing canonical pathways in N6 (WT) cells during the transition between passage 2 and passage 10. While the wild-type cells did not have any significant changes in cellular pathways (except three, A), several pathways including the human embryonic stem cell pluripotency in Rest+/− (B) and Nanog embryonic stem cell pluripotency pathway changed in Rest−/− cells (C). (D, E and F) Validation of the results of genome-wide analyses in Rest+/− and Rest−/− ESCs. (D) Western blotting assays to measure REST protein levels (left panel: Western blot, right panels: quantification of western blot) and the core pluripotency factors Oct4, Sox2, and Nanog (left panel: Representative Western blot, right panels: quantification of three western blots for Rest+/− and four western blots for Rest−/−) in cells cultured for 2, 5, and 10 passages. Actin was used as a loading control. Error bars are standard error of means. Quantification of western blots was performed using Odyssey V3.0 software and was plotted after normalization against actin. REST protein levels were elevated in Rest+/− cells over passages but, it remained unchanged in the wild-type and Rest−/− cells. Whereas the wild-type cells maintained high levels of the core self-renewal factors Oct4, Sox2, and Nanog throughout the passages, their levels were significantly lower during the early passages of Rest +/− and Rest−/− cells and increased with passage. Nanog levels increased the most in Rest−/− cells. (E) Relative expression levels of Rest in N9 (Rest+/−) and N8 (Rest^−/−^) cells (confirmed with three independent primer sets) are shown after normalization with N6 (WT) cells grown for equivalent passage. Passage numbers are shown on the x-axis. p-values are shown over the bars. Gapdh was used as control. Error bars are standard error of means (n = 3). (F) Relative expression levels of pluripotency factors (Nanog, Oct4 and Sox2) in N9 (Rest+/−) and N8 (Rest^−/−^) cells are shown after normalization with N6 (WT) cells grown for equivalent passages. Passage numbers are shown on the x-axis. The p-values are shown over the bars. Gapdh was used as control. Error bars are standard error of means (n = 3).

To determine whether there were changes in the ESC-specific canonical pathways in N6 (WT), N9 (Rest^+/−^) and N8 (Rest^−/−^) cells during the transition between passage 2 and passage 10, we used the IPA program to analyze the potential pathway changes in these cells. The results indicated that the wild-type cells did not have any significant changes (except three involving regulation of actin-based motility by Rho, role of tissue factor in cancer and B-cell receptor signaling) in cellular pathways during the transition from passage 2 to passage 10 (Figure 2A). In contrast, several pathways changed during the prolonged culture of the N9 (Rest^+/−^) (Figure 2B) and N8 (Rest^−/−^) cells (Figure 2C). Importantly, the pathway representing the human embryonic stem cells in N9 (Rest^+/−^) cells (p = 1.55E-06) and the role of Nanog in mammalian embryonic stem cell pluripotency was the second most-altered pathway in the Rest^−/−^ cells (p = 2E-06) (the top 5 changed pathways, with p-values, are shown in Table S4).

### Restoration of the impaired self-renewal and pluripotency of Rest+/− and Rest−/− AB-1 ESCs parallels increased REST expression in Rest+/− and increased pluripotency factors in both Rest+/− and Rest−/− cells

To validate the genome-wide analysis, we used Western blotting assays to measure REST protein levels (Figure 2D: left panel: Western blot, right panels: quantification of western blot) and the core pluripotency factors Oct4, Sox2, and Nanog in cells cultured for 2, 5, and 10 passages (Figure 2D; left panel: Western blot, right panels: quantification of western blot). Surprisingly, REST protein levels were elevated in N9 (Rest^+/−^) cells with increased passage whereas, it remained unchanged in the N6 (WT) and N8 (Rest^−/−^) cells (Figure 2D and Figure S2), as expected. Consistent with previous reports [30], [31], [32], [33], [34], even though no REST protein can be made in N8 (Rest^−/−^) cell line, we observed an increase in the transcript levels of the truncated Rest gene during this recovery of impaired self-renewal (Figure 2E). As described above, one of the possibilities is that there is a yet-unknown homeostatic mechanism that attempts to upregulate Rest expression when the Rest transcript levels are lower as in Rest+/− cells compared with wild-type cells.

Whereas the wild-type cells maintained high levels of the core self-renewal factors throughout the passages, the protein levels of Nanog (Figure S3), Oct4 (Figure S4) and Sox2 (Figure S5) were significantly lower during the early passages of N9 (Rest^+/−^) and N8 (Rest^−/−^) cells compared to the parental control ESCs (Figure 2D). However, the levels of these factors (Nanog, Oct4 and Sox2) in the N9 (Rest^+/−^) and in N8 (Rest^−/−^) cells increased with passage, such that by passage 10, their levels were equivalent to those in the wild-type control ESCs. Interestingly, the levels of Nanog in the N8 (Rest^−/−^) cells significantly increased during the transition from early to late passage, to a level about 2-fold higher than that in the control cells. The increased protein levels of the core pluripotency factors during their transition from passage 2 to passage 10 in Rest^−/−^ cells also paralleled in the increase in their corresponding transcript levels (Figure 2F). Thus, these results suggested that the elevated levels of pluripotency factors, particularly Nanog, played a critical role in the recovery of self-renewal of ESCs in the complete absence of REST protein. Thus, there appeared to be an intricate interaction between REST and the core pluripotency factors, including the Nanog pathway, in which the loss of REST is compensated by the pluripotency factors (see Discussion).

### Extracellular cues provided by feeder cells compensate for REST-mediated loss of self-renewal and pluripotency in AB-1 and E14Tg2a.4 ESCs

**Figure 3:**
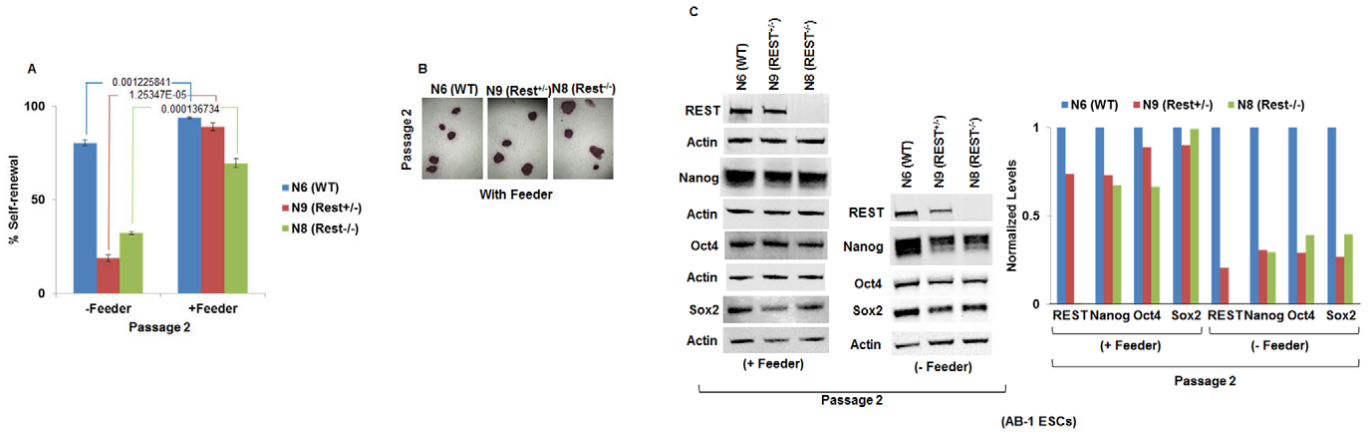
Extracellular cues provided by feeder cells compensate for REST-mediated loss of self-renewal and pluripotency in AB-1 ESCs. (A) Alkaline phosphatase based self-renewal assays of AB-1 parental, Rest+/− and Rest−/− ESCs in the presence of LIF but in the presence or absence of feeder cells shows that Rest+/− and Rest−/− cells have lower self-renewal efficiency compared to the parental line in the absence of feeder cells, but no large difference in the presence of feeder cells. The numbers under the lines show p-values (n = 3). Error bars are standard error of means. (B) Representative pictures of AP-stained colonies of parental and Rest−/− cells in the presence of feeder layer. (C) Western blotting assays to detect level of REST and the core self-renewal regulators Oct4, Sox2, and Nanog (left panel: Western blot, right panels: quantification of western blot) confirmed that the feeder cells compensated for the loss of self-renewal and pluripotency in Rest+/− and Rest−/− AB-1 ESCs. Experiments were performed as described in Figure 2D.

The previous reports using Rest-null AB-1 cells cultured in the presence of feeder cells indicated that REST plays no role in the maintenance of ESC self-renewal and pluripotency [27]. To determine whether the presence of feeder cells in these experiments was responsible for the difference between their results and the results of our experiments performed without feeder cells (described above), we cultured the N6 (WT), N9 (Rest^+/−^) and N8 (Rest^−/−^) AB-1 cells in the presence and absence of feeder cells. In contrast to our results obtained in the absence of feeder cells (Figure 1 A and B), the presence of feeder cells resulted in no large difference in self-renewal efficiency between the N6 (WT), N9 (Rest^+/−^) and N8 (Rest^−/−^), as determined by AP assay (Figure 3A: quantification of AP self-renewal assay; Figure 3B: representative pictures of AP-stained colonies). Further Western blotting assays to detect the core self-renewal regulators Oct4, Sox2, and Nanog (Figure 3C; left panel: Western blot, right panels: quantification of western blot) confirmed that the feeder cells compensated for the loss of self-renewal and pluripotency in N9 (Rest^+/−^) and N8 (Rest^−/−^) AB-1 ESCs. Interestingly, REST protein levels in N9 (Rest^+/−^) cells when cultured in presence of feeder cells is similar to that of N9 (Rest+/−) cells cultured without feeder at passage 10.

**Figure 4:**
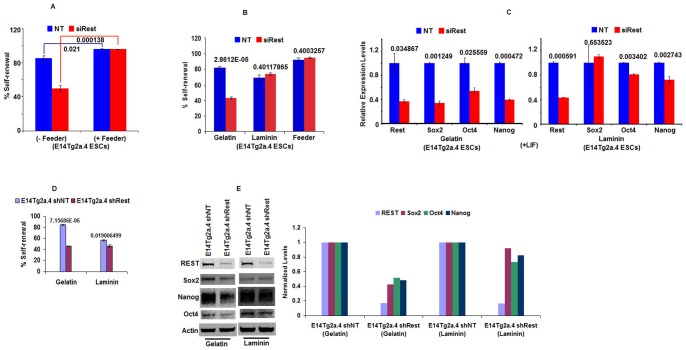
Extracellular cues provided by feeder cells compensate for REST-mediated loss of self-renewal and pluripotency in E14Tg2a.4 ESCs. (A,B) Rest Mediated pluripotency is influenced by extracellular environment in E14Tg2a.4 ESCs. (A) MEF feeder cells and (B) the extracellular matrix component, laminin, countered lowered self-renewal in E14Tg2a.4 ESCs treated with siRNA mediated knockdown of REST (siRest). Percentages of self-renewing colonies in ES, NT and siRest treated cells after alkaline phosphatase assay are shown for each conditions (as labeled on x-axis). Both MEF and laminin condition resulted in maintenance of self-renewal compared to gelatin condition after siRest treatment. Number above bars are p-values (n = 3). Error bars are standard error of means. (C) Laminin maintains the expression of pluripotent markers in siRest treated E14Tg2a.4 ESCs. ESCs transfected with siRest or NT were plated on gelatin- or laminin-coated surfaces and were analyzed by QRT-PCR assays to determine the expression levels of Sox2, Nanog, and Oct4 transcripts. Number above bars are p-values (n = 3). Error bars are standard error of means. (D,E) Laminin counters lowered self-renewal and pluripotency due to loss of REST in shRest-mediated stable knock-down of REST in ESCs. (D) shRest lowered self-renewal when cells were cultured on gelatin but not on laminin. Percentages of self-renewing colonies in shRest and NT cells are shown for each conditions (as labeled on x-axis). Number above bars are p-values (n = 3). Error bars are standard error of means. (E) Stable shRest lines, when compared to shNT lines, of ESCs showed decreased levels of REST proteins when the cells were cultured either on gelatin or on laminin; however, the levels of Sox2, Nanog, and Oct4 were decreased only when cells were cultured on gelatin but not laminin.

To determine if a similar mechanism operated in the E14Tg2a.4 ESC line, we performed knock-down experiments in these cells using siRest [or nontargeting (NT) control siRNA], cultured them in the presence or absence of feeder cells, and determined their self-renewal efficiency with AP assay (Figure 4A). The results indicated that the lower self-renewal observed in the siRest-treated cells cultured in the absence of feeder cells could be countered by culturing the cells with feeder cells. Further analysis indicated that laminin, an extracellular matrix component, could counter lowered self-renewal in ESCs that were treated with siRest (Figure 4B). Analysis by quantitative RT-PCR (qRT-PCR) of the transcript levels of the cells cultured without feeder cells or with laminin further indicated that while the siRest treatment lowered Rest levels in cells cultured in either condition, the levels of the self-renewal regulators Oct4, Sox2, and Nanog were lowered extensively in the cells cultured without feeder cells and to a lesser extent in the cells cultured with laminin (Figure 4C). Previous studies showed that REST maintained pluripotency of E14Tg2a.4 cultured on gelatin by repressing the transcription of miRNA-21 [25]. Therefore, we evaluated the status of miRNA-21 levels in cells after siRest treatment and cultured either on gelatin or on laminin surfaces. MiRNA-21 levels were elevated in siRest-treated cells but only when the cells were cultured on gelatin coated surfaces and not when cells were cultured on laminin coated surfaces (supplementary Figure S6). These results suggest that culturing cells on laminin triggered a yet unknown mechanism that uncoupled the REST-miRNA-21 axis.

To determine whether laminin could counter the REST-mediated loss of self-renewal and pluripotency in long-term culture of E14Tg2a.4 ESCs, as compared to the immediate effects seen in the transient siRest-mediated knock-down experiments described above, we purchased new stock of E14Tg2a.4 ESCs to verify that our earlier results did not arise because of unforeseen problems with our cell line during laboratory culture. Further analysis indicated that the new cells were of normal karyotype (Figure S7). We then generated stable shRest and NT control ESC lines. After selecting puromycin-resistant and GFP-positive (by FACS) clones, we performed self-renewal assays (Figure 4D) and determined the protein levels of REST, Oct4, Sox2, and Nanog (Figure 4E; left panel: Western blot, right panels: quantification of western blot). The results supported the conclusion reached by siRest experiments and showed that while shRest-treated cells lowered the REST protein levels in cells cultured either on gelatin or on laminin, the self-renewal efficiency and the levels of corresponding self-renewal and pluripotenmcy regulators were substantially lower when the cells were cultured on gelatin, but a minimal reduction was observed when cells were cultured on laminin. Thus, the lowered self-renewal caused by lowered REST levels in E14Tg2a.4 ESCs, as in AB-1 ESCs, could be countered by culturing the cells with laminin. The results indicate that the loss of REST-mediated ESC pluripotency can be countered by feeder cells or extracellular matrix components and may be seen only when the ESCs are cultured in the absence of these extracellular cues.

### Stable knockdown of REST in E14Tg2a.4 ESCs causes decreased self-renewal and pluripotency and increased expression of differentiation markers under feeder-free conditions

Previously, we had used two different Rest+/− lines and siRNA-mediated knockdown of REST in E14Tg2a.4 ESCs. To determine whether stable knockdown of REST in these cells made any difference in the role of REST in ESC pluripotency, we generated stable shRest and NT control ESC lines of E14Tg2a.4 cells. First, we performed self-renewal assays. The E14Tg2a.4 cells showed decreased self-renewal, as reported earlier [25] (Figure 4D) (see Figure S8 for representative AP-stained colonies and calculations for self-renewal efficiency). The western blot analysis results (Figure 4E; left panel: Western blot, right panels: quantification of western blot) is consistent with that of self-renewal assay shown in Figure 4D. The REST levels showed distinct decreases due to shRest treatment. In the shRest E14Tg2a.4 cells, loss of REST resulted in lowered levels of Sox2, Oct4, and Nanog protein as compared to control.

To determine whether the self-renewal status of these cells corresponded to the expression of differentiation lineage markers, we performed qRT-PCR assays. E14Tg2a.4 cells treated with shRest showed significant increases in the expression of lineage markers representing the ectoderm, trophectoderm, mesoderm, and endoderm that were concomitant to their decreased self-renewal and pluripotency (Figure S9).

## Discussion

### Conditional role of REST as an ESC pluripotency maintenance factor

This work attempts to resolve the current discrepancies in the various published reports about the role of REST in the maintenance of ESC self-renewal and pluripotency. Here we show that REST, a maintenance factor, maintains self-renewal and pluripotency in a culture condition and cell-dependent manner. Loss of self-renewal and pluripotency due to loss of REST in the shRest lines of E14Tg2a.4, which was originally generated as a feeder-independent line, can be seen in cells propagated for many generations. Loss of REST in AB-1 cells, which was generated as a feeder-dependent line, shows REST-dependent maintenance of self-renewal when they are shifted to feeder-free culture conditions, but only during early passages. This impaired self-renewal and pluripotency is then restored in late passage cells, presumably owing to a culture condition-dependent response. However, the exact mechanism or mechanisms that lead to this response is unknown. One mechanism could be through a compensatory response, in which majority of cells respond to the selective pressure by capturing alternate pathways, such as increased Nanog pathway, to regain their self-renewal and pluripotency. Such a potential connection between Nanog and REST is supported by the observation that the greatest increase in the Nanog protein levels was also seen in the V6.5 ESC line upon loss of REST (29). In addition, REST has been shown to be in complex with Nanog by protein-protein interaction [33], [34]. And, loss of one component could be compensated by increased levels of another component of protein complexes that regulate important biological functions. For example, loss of Rpb4 can be countered by increased levels of another component Rpb7 in the RNA polymerase II protein complex [39]. Thus, it is conceivable that the decreased ESC self-renewal/pluripotency because of loss of REST is recovered, at least in part, due to a compensatory role of Nanog and the Nanog-pluripotency pathway. However, another mechanism could be through the selective expansion of minor population of cells containing high levels of pluripotency factors and high capacity of self-renewal. These cells, which constituted a minor population at early passage, are selected to become a major population after prolonged culturing. Thus, the explanation for the observed restoration of pluripotency and self-renewal may be due to expansion of specific cell population or a compensatory response or both.

### Extracellular cues influence ESC Pluripotency

Our observations presented here support an interesting hypothesis that REST-mediated mESC pluripotency depends on both the intracellular regulatory network and the extracellular environment. This might explain why some studies have found that REST plays a role in the maintenance of mESC pluripotency while others have not (see Introduction). It is likely that the regulatory pathways of mESC pluripotency that are regulated by factors other than REST are also maintained in equilibrium with environmental cues.

## Conclusions

Here we provide evidence that REST maintains self-renewal and pluripotency of ESCs in a context-dependent fashion and that ESC pluripotency should be evaluated in a context-dependent manner.

## Supporting Information

Figure S1 **Impaired self-renewal efficiency is restored after prolonged culturing.** Alkaline phosphatase based self-renewal assay for WT, Rest+/− and Rest−/− cells cultured in parallel for 2 and 10 passages. Top: Scanned image of self-renewal assay plates. Sample name are labeled on the left side and passage numbers are at the bottom of the images. Bottom: Table for total number of colonies in self-renewal plates, differentiating (white), self-renewing colonies (pink) and percent self-renewal is shown.
(TIF)

Figure S2 **REST protein levels is elevated in Rest^+/−^ ESCs over prolonged culturing under self-renewing conditions.** Western blot analysis for REST in whole cell lysate from N6 (WT), N9 (Rest^+/−^) and N8 (Rest^−/−^) ESCs after passaging without feeder layer but in presence of LIF is shown. Actin for same membrane is shown on the left. Whole blot image of three membranes are shown. Lanes are numbered on top of each blot. The table at the bottom shows the sample names and their passage numbers.
(TIF)

Figure S3 **Nanog protein levels is elevated in Rest^+/−^ and Rest^−/−^ ESCs over prolonged culturing under self-renewing conditions.** Western blot analysis for Nanog in whole cell lysate from N6 (WT), N9 (Rest^+/−^) and N8 (Rest^−/−^) ESCs after passaging without feeder layer but in presence of LIF is shown. Actin for same membrane is shown on the left. Whole blot image of three membranes are shown. Lanes are numbered on top of each blot. The table at the bottom shows the sample names and their passage numbers.
(TIF)

Figure S4 **Oct4 protein levels is elevated in Rest^+/−^ and Rest^−/−^ ESCs over prolonged culturing under self-renewing conditions.** Western blot analysis for Oct4 in whole cell lysate from N6 (WT), N9 (Rest^+/−^) and N8 (Rest^−/−^) ESCs after passaging without feeder layer but in presence of LIF is shown. Actin for same membrane is shown on the left. Whole blot image of three membranes are shown. Lanes are numbered on top of each blot. The table at the bottom shows the sample names and their passage numbers.
(TIF)

Figure S5 **Sox2 protein levels is elevated in Rest^+/−^ and Rest^−/−^ ESCs over prolonged culturing under self-renewing conditions.** Western blot analysis for Sox2 in whole cell lysate from N6 (WT), N9 (Rest^+/−^) and N8 (Rest^−/−^) ESCs after passaging without feeder layer but in presence of LIF is shown. Actin for same membrane is shown on the left. Whole blot image of three membranes are shown. Lanes are numbered on top of each blot. The table at the bottom shows the sample names and their passage numbers.
(TIF)

Figure S6 **siRNA mediated nockdown of REST results in elevated miRNA-21 levels when cultured on gelatin, but not laminin, coated surface.** Quantitative real time reverse-transcription PCR analysis of miR-21 levels after siRNA mediated knockdown of REST. Cells were cultured on either gelatin or laminin surfaces for 3 days. The miR-21 levels were normalized against 5S rRNA levels in their respective samples and siNT control. The values above the bars are p-values (n = 3). Error bars are standard error of means.
(TIF)

Figure S7 **G-banding based karyotype analysis of E14Tg2a.4 ESCs.** Representative slide for G-band karyotype analysis shown normal karyotype for E14Tg2a.4 ESCs (from Bay Genomics) is shown. Chromosome numbers are shown under each chromosome.
(TIF)

Figure S8 **Impaired self-renewal due to loss of Rest in E14Tg2a.4.** Alkaline phosphatase based self-renewal assay for shRNA mediated knockdown of Rest in E14Tg2a.4 is shown. Left: Scanned image of self-renewal assay plates. Sample name are labeled on the left side. Center: Schematic representation of self-renewal plates with treatment is shown [shNT (control) and shRest]. Right: Table for total number of colonies in self-renewal plates, self-renewing colonies (pink) and percent self-renewal is shown.
(TIF)

Figure S9 **Stable knockdown of REST in E14Tg2a.4 ESCs causes increased expression of differentiation markers under feeder-free conditions.** Quantitative real time reverse-transcription PCR analysis of lineage markers after shRNA mediated knockdown of Rest in E14Tg2a.4. The results were normalized against Gapdh and shNT control. The values above the bars are p-values (n = 3). Error bars are standard error of means. Ectoderm (orange), trophoectoderm (blue), Mesoderm (green) and endoderm (maroon).
(TIF)

Table S1 **Genes with maximum fold change in N9 (Rest^+/−)^ (passage 2 vs. passage 10) (−ve values: downregulated; +ve values: Upregulated).**
(DOCX)

Table S2 **Genes with maximum fold change in N8 (Rest^−/−^) (passage 2 vs. passage 10) (−ve values: downregulated; +ve values: Upregulated).**
(DOCX)

Table S3 **Comparison of FunGenES database to the 50 up-regulated or down-regulated genes in N9 (Rest+/−) and N8 (Rest−/−) AB-1 ESCs during the transition from passage 2 to passage 10.**
(DOCX)

Table S4 **List of top five canonical pathways being altered in N6 (WT), N9 (Rest+/−), and N8 (Rest−/−) ESCs after prolonged culturing.**
(DOCX)
