# Supplementary figures and images for: Dynamic Status of REST in the Mouse ESC Pluripotency Network
Source: PLoS One. 2012 Aug 28;7(8):e43659. doi: 10.1371/journal.pone.0043659 (PMC3429488; doi:10.1371/journal.pone.0043659)

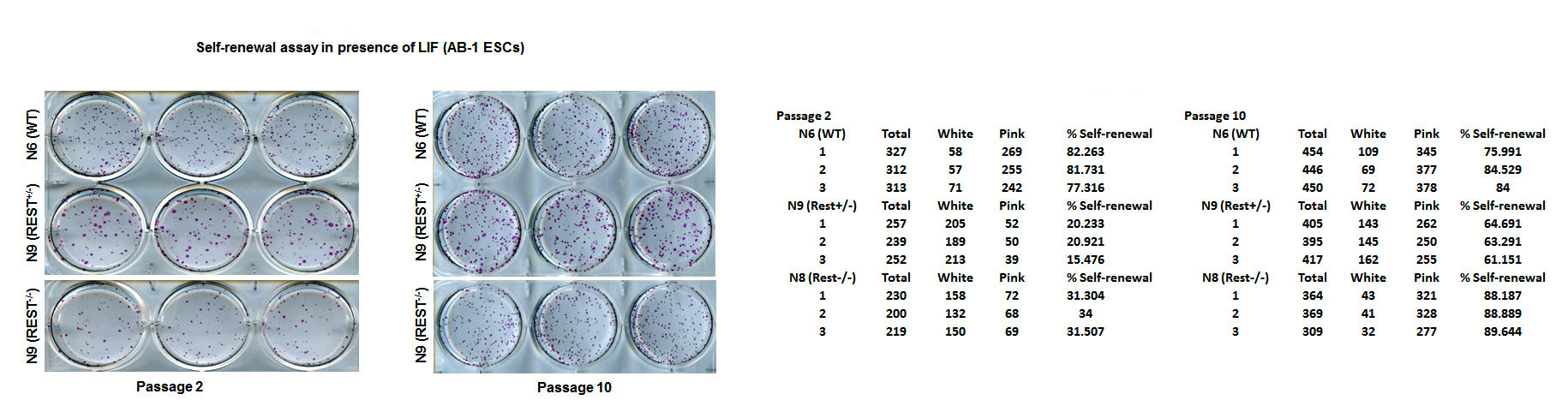

Supplement: Figure S1 — Impaired self-renewal efficiency is restored after prolonged culturing. Alkaline phosphatase based self-renewal assay for WT, Rest+/− and Rest−/− cells cultured in parallel for 2 and 10 passages. Top: Scanned image of self-renewal assay plates. Sample name are labeled on the left side and passage numbers are at the bottom of the images. Bottom: Table for total number of colonies in self-renewal plates, differentiating (white), self-renewing colonies (pink) and percent self-renewal is shown. (TIF) [file pone.0043659.s001.tif]

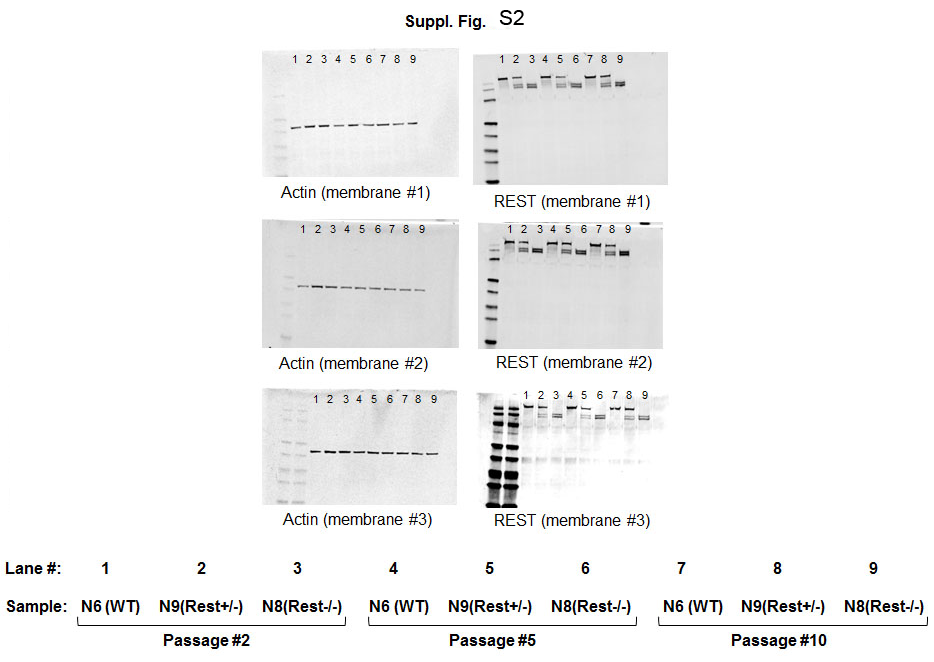

Supplement: Figure S2 — REST protein levels is elevated in Rest+/− ESCs over prolonged culturing under self-renewing conditions. Western blot analysis for REST in whole cell lysate from N6 (WT), N9 (Rest+/−) and N8 (Rest−/−) ESCs after passaging without feeder layer but in presence of LIF is shown. Actin for same membrane is shown on the left. Whole blot image of three membranes are shown. Lanes are numbered on top of each blot. The table at the bottom shows the sample names and their passage numbers. (TIF) [file pone.0043659.s002.tif]

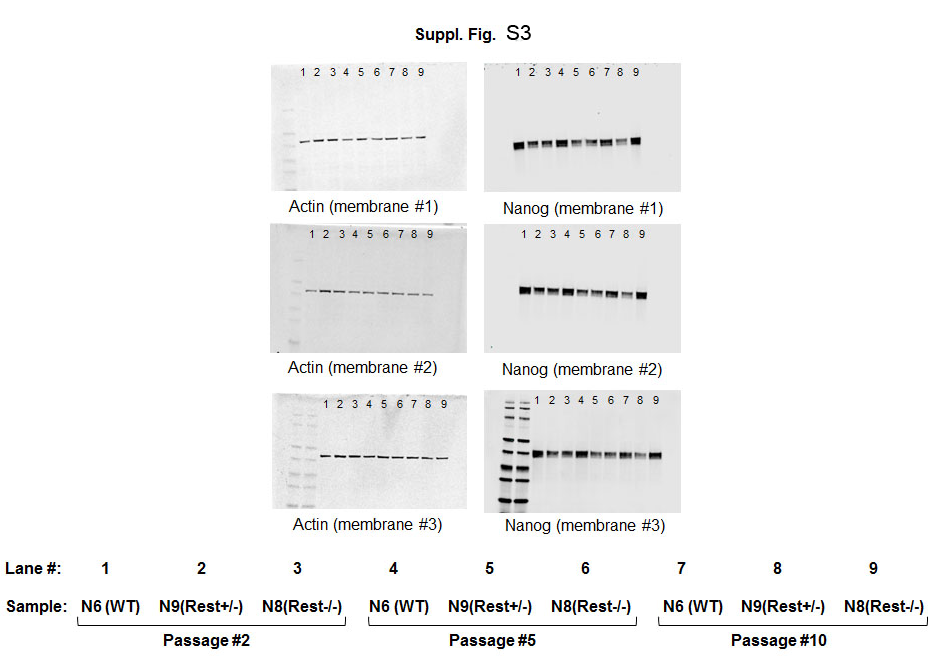

Supplement: Figure S3 — Nanog protein levels is elevated in Rest+/− and Rest−/− ESCs over prolonged culturing under self-renewing conditions. Western blot analysis for Nanog in whole cell lysate from N6 (WT), N9 (Rest+/−) and N8 (Rest−/−) ESCs after passaging without feeder layer but in presence of LIF is shown. Actin for same membrane is shown on the left. Whole blot image of three membranes are shown. Lanes are numbered on top of each blot. The table at the bottom shows the sample names and their passage numbers. (TIF) [file pone.0043659.s003.tif]

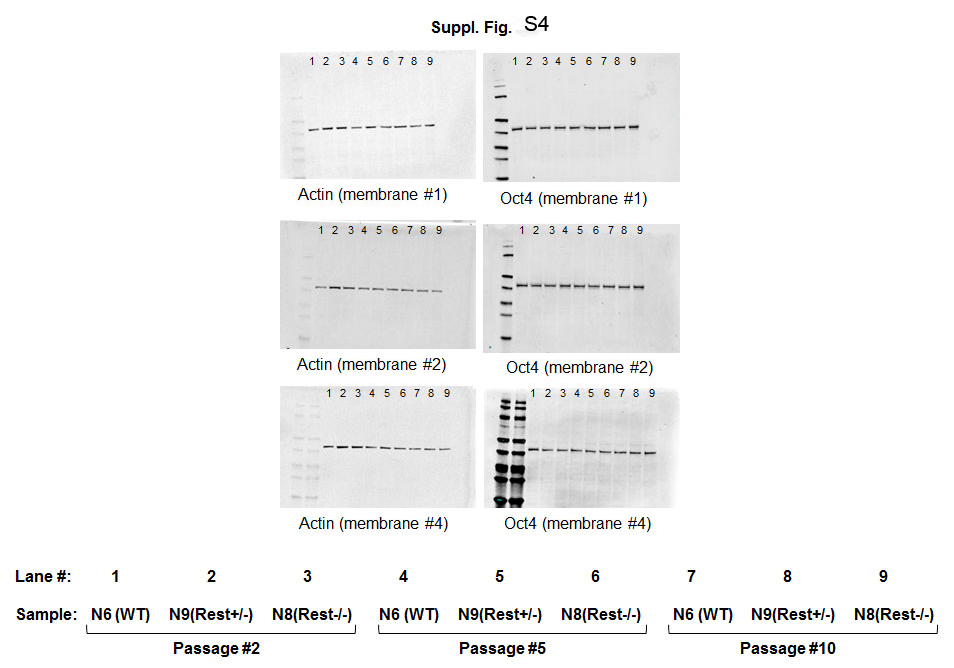

Supplement: Figure S4 — Oct4 protein levels is elevated in Rest+/− and Rest−/− ESCs over prolonged culturing under self-renewing conditions. Western blot analysis for Oct4 in whole cell lysate from N6 (WT), N9 (Rest+/−) and N8 (Rest−/−) ESCs after passaging without feeder layer but in presence of LIF is shown. Actin for same membrane is shown on the left. Whole blot image of three membranes are shown. Lanes are numbered on top of each blot. The table at the bottom shows the sample names and their passage numbers. (TIF) [file pone.0043659.s004.tif]

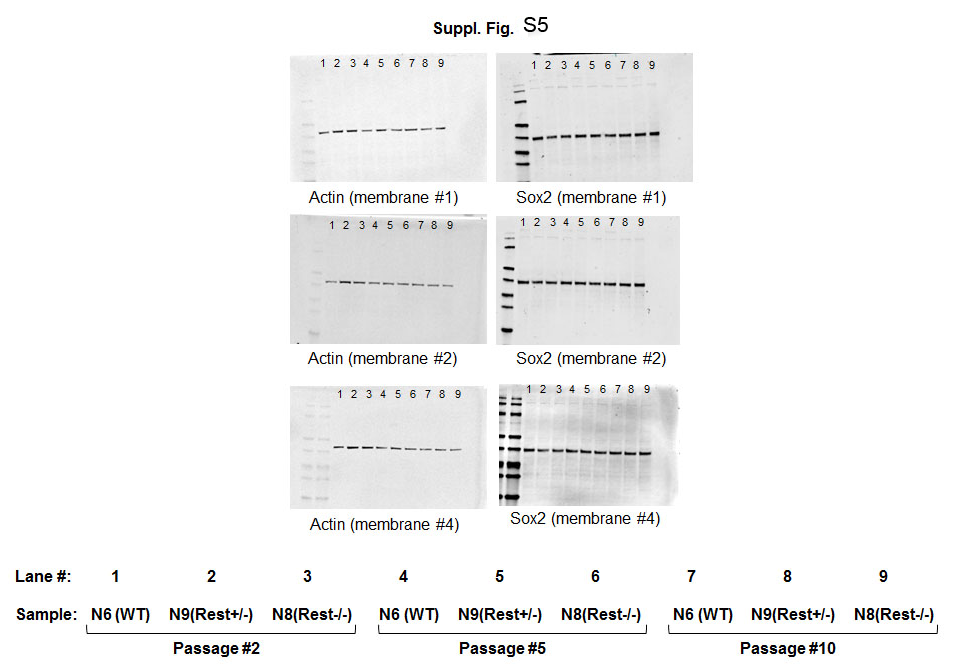

Supplement: Figure S5 — Sox2 protein levels is elevated in Rest+/− and Rest−/− ESCs over prolonged culturing under self-renewing conditions. Western blot analysis for Sox2 in whole cell lysate from N6 (WT), N9 (Rest+/−) and N8 (Rest−/−) ESCs after passaging without feeder layer but in presence of LIF is shown. Actin for same membrane is shown on the left. Whole blot image of three membranes are shown. Lanes are numbered on top of each blot. The table at the bottom shows the sample names and their passage numbers. (TIF) [file pone.0043659.s005.tif]

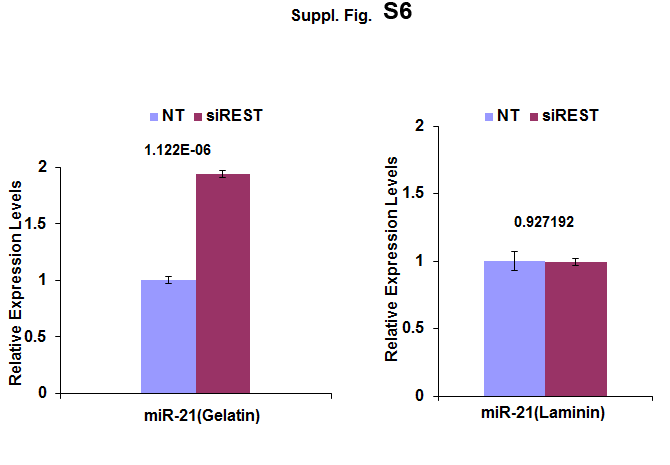

Supplement: Figure S6 — siRNA mediated nockdown of REST results in elevated miRNA-21 levels when cultured on gelatin, but not laminin, coated surface. Quantitative real time reverse-transcription PCR analysis of miR-21 levels after siRNA mediated knockdown of REST. Cells were cultured on either gelatin or laminin surfaces for 3 days. The miR-21 levels were normalized against 5S rRNA levels in their respective samples and siNT control. The values above the bars are p-values (n = 3). Error bars are standard error of means. (TIF) [file pone.0043659.s006.tif]

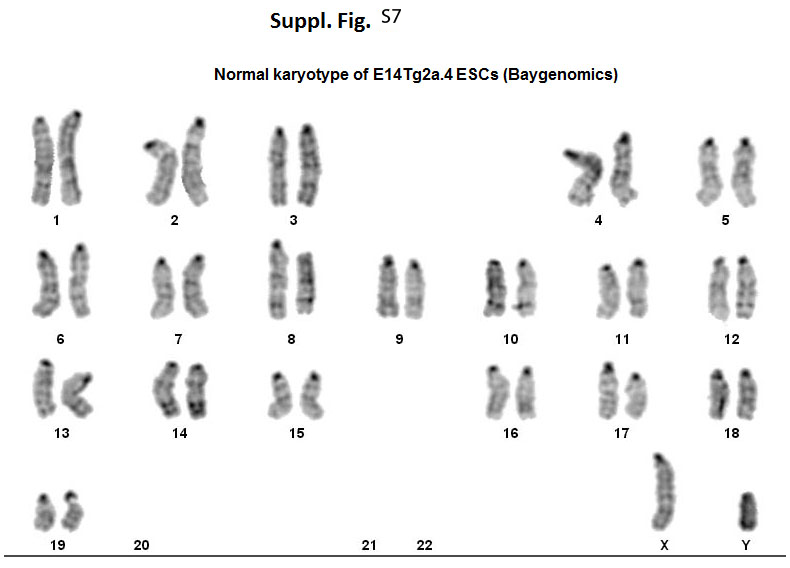

Supplement: Figure S7 — G-banding based karyotype analysis of E14Tg2a.4 ESCs. Representative slide for G-band karyotype analysis shown normal karyotype for E14Tg2a.4 ESCs (from Bay Genomics) is shown. Chromosome numbers are shown under each chromosome. (TIF) [file pone.0043659.s007.tif]

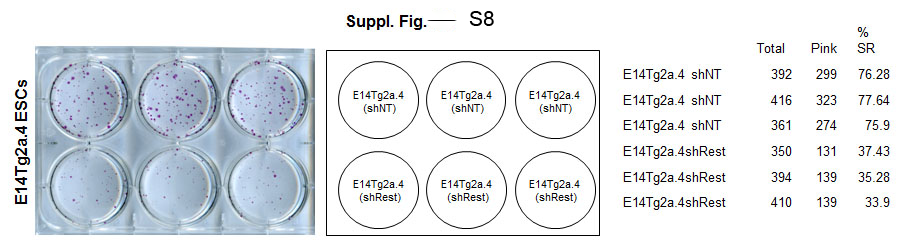

Supplement: Figure S8 — Impaired self-renewal due to loss of Rest in E14Tg2a.4. Alkaline phosphatase based self-renewal assay for shRNA mediated knockdown of Rest in E14Tg2a.4 is shown. Left: Scanned image of self-renewal assay plates. Sample name are labeled on the left side. Center: Schematic representation of self-renewal plates with treatment is shown [shNT (control) and shRest]. Right: Table for total number of colonies in self-renewal plates, self-renewing colonies (pink) and percent self-renewal is shown. (TIF) [file pone.0043659.s008.tif]

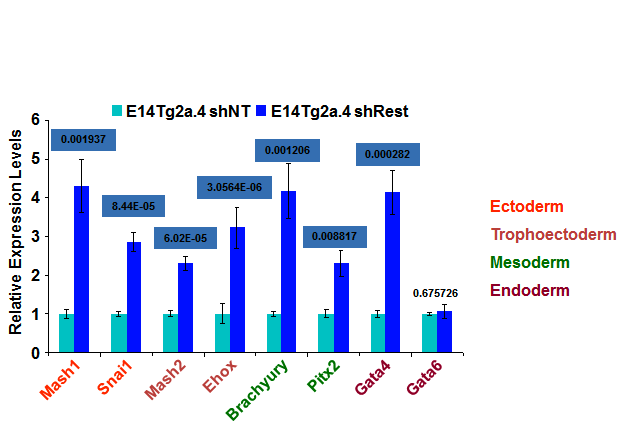

Supplement: Figure S9 — Stable knockdown of REST in E14Tg2a.4 ESCs causes increased expression of differentiation markers under feeder-free conditions. Quantitative real time reverse-transcription PCR analysis of lineage markers after shRNA mediated knockdown of Rest in E14Tg2a.4. The results were normalized against Gapdh and shNT control. The values above the bars are p-values (n = 3). Error bars are standard error of means. Ectoderm (orange), trophoectoderm (blue), Mesoderm (green) and endoderm (maroon). (TIF) [file pone.0043659.s009.tif]
