# Supplementary material for: Dynamic Status of REST in the Mouse ESC Pluripotency Network
Source: PLoS One. 2012 Aug 28;7(8):e43659. doi: 10.1371/journal.pone.0043659 (PMC3429488; doi:10.1371/journal.pone.0043659)
Supplement: Table S1 — Genes with maximum fold change in N9 (Rest+/−) (passage 2 vs. passage 10) (−ve values: downregulated; +ve values: Upregulated). (DOCX) [file pone.0043659.s010.docx]

**Table S1. Genes with maximum fold change in N9 (Rest^+/-)^ (passage 2 vs. passage 10) (-ve values: downregulated; +ve values: Upregulated).**

| S. No. | Fold Change | Gene Symbol |  | S. No. | Fold Change | Gene Symbol |
| --- | --- | --- | --- | --- | --- | --- |
| 1 | -23.083887 | T |  | 1 | 5.5707846 | 1700019A02Rik |
| 2 | -15.5141325 | Pmp22 |  | 2 | 4.781368 | Otx2 |
| 3 | -15.135443 | Tgfb2 |  | 3 | 4.4927025 | B4galnt2 |
| 4 | -14.470084 | Cryab |  | 4 | 4.248941 | Enpp3 |
| 5 | -13.161363 | Wisp1 |  | 5 | 4.175731 | Gna14 |
| 6 | -12.857802 | Col4a5 |  | 6 | 3.9493182 | Aldh1l2 |
| 7 | -12.723749 | Ppbp |  | 7 | 3.9133155 | Slc27a2 |
| 8 | -12.402301 | Igfbp5 |  | 8 | 3.8522875 | Stc2 |
| 9 | -12.250599 | Sprr2a |  | 9 | 3.8307562 | Pnma5 |
| 10 | -11.789005 | Prss23 |  | 10 | 3.826379 | Calca |
| 11 | -11.654015 | Acta2 |  | 11 | 3.743874 | Liph |
| 12 | -11.035929 | Prss23 |  | 12 | 3.5476255 | Pecam1 |
| 13 | -10.799132 | Cd44 |  | 13 | 3.4953597 | Vldlr |
| 14 | -10.396732 | Nts |  | 14 | 3.4251425 | Prkar2b |
| 15 | -10.014699 | Gpr177 |  | 15 | 3.3944635 | 9330199F22Rik |
| 16 | -9.886106 | Col11a1 |  | 16 | 3.3912294 | Spna1 |
| 17 | -9.590172 | Cdx1 |  | 17 | 3.276166 | Cnr1 |
| 18 | -9.165168 | Acta1 |  | 18 | 3.2544613 | Myb |
| 19 | -8.9776745 | Cdkn2b |  | 19 | 3.222986 | Pycr1 |
| 20 | -8.876366 | Ets1 |  | 20 | 3.206486 | Serpini1 |
| 21 | -8.854878 | Serpinb9 |  | 21 | 3.1654859 | Rbm44 |
| 22 | -8.281067 | Tnfrsf19 |  | 22 | 3.120772 | Trib3 |
| 23 | -7.91072 | Npnt |  | 23 | 3.0832903 | Neurod1 |
| 24 | -7.835136 | Col3a1 |  | 24 | 3.0817885 | Car12 |
| 25 | -7.7288866 | Thbs1 |  | 25 | 3.0205123 | Acss1 |
| 26 | -7.4286437 | Axl |  | 26 | 2.994854 | Dmc1 |
| 27 | -7.418331 | Gsn |  | 27 | 2.9690387 | Atp12a |
| 28 | -7.416554 | Col1a1 |  | 28 | 2.945258 | D630023F18Rik |
| 29 | -7.30174 | Anxa5 |  | 29 | 2.9204898 | Six6os1 |
| 30 | -6.977015 | Cyp1b1 |  | 30 | 2.9028478 | 2410076I21Rik |
| 31 | -6.975855 | Sema3c |  | 31 | 2.8839614 | Mt1 |
| 32 | -6.840909 | Tagln |  | 32 | 2.8801806 | C530008M17Rik |
| 33 | -6.8346195 | Vnn1 |  | 33 | 2.8710256 | Irs4 |
| 34 | -6.7867756 | Bmp2 |  | 34 | 2.847939 | Nupr1 |
| 35 | -6.785845 | Vim |  | 35 | 2.8371496 | Enpp3 |
| 36 | -6.6595426 | Vgll3 |  | 36 | 2.8306286 | Gm364 |
| 37 | -6.521001 | Irs1 |  | 37 | 2.8227527 | Tek |
| 38 | -6.5067997 | Tnc |  | 38 | 2.7901282 | Chd9 |
| 39 | -6.5004544 | Cav1 |  | 39 | 2.767699 | Luzp4 |
| 40 | -6.429511 | 4-Sep |  | 40 | 2.7600803 | Arid5b |
| 41 | -6.3525066 | Hmga2 |  | 41 | 2.7515929 | Aff3 |
| 42 | -6.30995 | Cald1 |  | 42 | 2.7507863 | Wscd1 |
| 43 | -6.265611 | Fosl2 /// LOC634417 |  | 43 | 2.7464476 | Nxf3 |
| 44 | -6.082397 | Crim1 |  | 44 | 2.7301311 | Sycp3 |
| 45 | -6.0649233 | Aplnr |  | 45 | 2.7237797 | Nrsn1 |
| 46 | -6.0514965 | LOC100048431///Actc1 |  | 46 | 2.7172194 | Chac1 |
| 47 | -6.0202312 | Dsp |  | 47 | 2.714582 | Zmat4 |
| 48 | -5.8392015 | Hoxa1 |  | 48 | 2.6694279 | AW551984 |
| 49 | -5.7392893 | Krt8 |  | 49 | 2.6479497 | Vldlr |
| 50 | -5.7347083 | Tnfsf9 |  | 50 | 2.6373138 | Insm1 |
