# Supplementary material for: Dynamic Status of REST in the Mouse ESC Pluripotency Network
Source: PLoS One. 2012 Aug 28;7(8):e43659. doi: 10.1371/journal.pone.0043659 (PMC3429488; doi:10.1371/journal.pone.0043659)
Supplement: Table S2 — Genes with maximum fold change in N8 (Rest−/−) (passage 2 vs. passage 10) (−ve values: downregulated; +ve values: Upregulated). (DOCX) [file pone.0043659.s011.docx]

**Table S2. Genes with maximum fold change in N8 (Rest^-/-^) (passage 2 vs. passage 10) (-ve values: downregulated; +ve values: Upregulated).**

| S. No. | Fold change | Gene Symbol |  | S. No. | Fold change | Gene Symbol |
| --- | --- | --- | --- | --- | --- | --- |
| 1 | -21.84479 | T |  | 1 | 20.60585 | Tex13 |
| 2 | -16.78483 | Prss23 |  | 2 | 14.662669 | Slc38a5 |
| 3 | -16.374355 | Efemp1 |  | 3 | 11.106372 | Lrrc31 |
| 4 | -15.634183 | Gbp2 |  | 4 | 9.135895 | Xaf1 |
| 5 | -14.106334 | Phldb2 |  | 5 | 7.739626 | Jam2 |
| 6 | -13.56542 | Krt8 |  | 6 | 6.5274396 | Ttc29 |
| 7 | -13.18991 | Sept4 |  | 7 | 6.2055283 | Klk1 |
| 8 | -12.148453 | Acta2 |  | 8 | 5.820763 | Gm364 |
| 9 | -11.810791 | Tagln |  | 9 | 5.7398624 | Slc47a1 |
| 10 | -11.416139 | Ets1 |  | 10 | 5.737749 | Nupr1 |
| 11 | -11.148488 | Prtg |  | 11 | 5.3643994 | 2410003l16Rik |
| 12 | -11.147794 | Krt18 |  | 12 | 5.2938766 | Inhbb |
| 13 | -10.6019 | Hmga2 |  | 13 | 5.258808 | Slc6a1 |
| 14 | -10.06967 | Fzd2 |  | 14 | 5.253405 | Mcf2 |
| 15 | -9.980914 | Anxa3 |  | 15 | 5.196783 | Tbx3 |
| 16 | -9.795373 | Cryab |  | 16 | 5.161948 | Prdm14 |
| 17 | -9.282981 | Pmp22 |  | 17 | 5.143332 | Lrrc34 |
| 18 | -9.129948 | Lgals7 |  | 18 | 5.0440493 | Tcl1 |
| 19 | -8.834637 | Tacstd2 |  | 19 | 5.0298667 | 4930422N03Rik |
| 20 | -8.75354 | Slc25a24 |  | 20 | 4.668111 | Ly75 |
| 21 | -8.57228 | Ghr |  | 21 | 4.634286 | Myo1f |
| 22 | -8.553414 | Acta1 |  | 22 | 4.5373034 | Klf4 |
| 23 | -8.2453785 | Gsn |  | 23 | 4.5098233 | nr0b1 |
| 24 | -7.9869537 | Fndc3c1 |  | 24 | 4.480279 | serpinb6c |
| 25 | -7.8407497 | Slc39a8 |  | 25 | 4.4787374 | Fetub |
| 26 | -7.680711 | Anxa1 |  | 26 | 4.394377 | Il2rg |
| 27 | -7.6783934 | Nefm |  | 27 | 4.3667183 | Serpinb1a |
| 28 | -7.5241923 | Sema3c |  | 28 | 4.20324 | Huwe1 |
| 29 | -7.464102 | Igfbp3 |  | 29 | 4.0999923 | Fcgr2b |
| 30 | -7.269701 | Nnat |  | 30 | 4.041402 | Hal |
| 31 | -7.2650266 | Cald1 |  | 31 | 4.009427 | Trps1 |
| 32 | -7.2471213 | Krt7 |  | 32 | 3.97487 | Crtac1 |
| 33 | -6.944619 | Fosl2 |  | 33 | 3.9020014 | Aldh1l2 |
| 34 | -6.910156 | Sp5 |  | 34 | 3.8935375 | Tet2 |
| 35 | -6.852006 | Lpp |  | 35 | 3.8772373 | Elovl7 |
| 36 | -6.746813 | Sema3e |  | 36 | 3.8541636 | Hormad2 |
| 37 | -6.702541 | Gpr177 |  | 37 | 3.8088644 | Padi4 |
| 38 | -6.562267 | Dsp |  | 38 | 3.6824899 | Prelid2 |
| 39 | -6.4040904 | Anxa5 |  | 39 | 3.6776524 | Fthl17 |
| 40 | -6.3072786 | Irs1 |  | 40 | 3.6687255 | Nxt2 |
| 41 | -6.2160835 | Igf2 |  | 41 | 3.646818 | Sfrp1 |
| 42 | -6.1675057 | Parva |  | 42 | 3.629957 | 1700019N12Rik |
| 43 | -5.9628997 | Lphn2 |  | 43 | 3.6121817 | Cobl |
| 44 | -5.925467 | Car4 |  | 44 | 3.6085744 | Dpysl4 |
| 45 | -5.89641 | Cited1 |  | 45 | 3.5666094 | Ceacam1 |
| 46 | -5.857145 | Nrk |  | 46 | 3.5552459 | Mitf |
| 47 | -5.8131857 | Fgf8 |  | 47 | 3.5438027 | Calca |
| 48 | -5.7824793 | Pitx2 |  | 48 | 3.4829404 | Adhfe1 |
| 49 | -5.758451 | Ptn |  | 49 | 3.4799004 | Slc25a36 |
| 50 | -5.7555995 | Il33 |  | 50 | 3.4670343 | Fgf4 |
