# Supplementary material for: Dynamic Status of REST in the Mouse ESC Pluripotency Network
Source: PLoS One. 2012 Aug 28;7(8):e43659. doi: 10.1371/journal.pone.0043659 (PMC3429488; doi:10.1371/journal.pone.0043659)
Supplement: Table S3 — Comparison of FunGenES database to the 50 up-regulated or down-regulated genes in N9 (Rest+/−) and N8 (Rest−/−) AB-1 ESCs during the transition from passage 2 to passage 10. (DOCX) [file pone.0043659.s012.docx]

**Table S3. Comparison of FunGenES database to the 50 up-regulated or down-regulated genes in N9 (Rest+/-) and N8 (Rest-/-) AB-1 ESCs during the transition from passage 2 to passage 10**

|  | N9 (Rest+/-) (Passage 10/2) | p-value | N8 (Rest-/-) (Passage 10/2) | p-value |
| --- | --- | --- | --- | --- |
| ES/EB6 (FunGenES database) | 37/50 (Upregulated) | 0.00999 | 39/50 (Upregulated) | 0.00163 |
| ES/EB6 (FunGenES database) | 46/50 (Downregulated) | 3.30E-13 | 45/50 (Downregulated) | 4.12E-12 |

ES: Self-renewing culture condition; EB6: Embryoid body 6 days
