# Supplementary material for: Dynamic Status of REST in the Mouse ESC Pluripotency Network
Source: PLoS One. 2012 Aug 28;7(8):e43659. doi: 10.1371/journal.pone.0043659 (PMC3429488; doi:10.1371/journal.pone.0043659)
Supplement: Table S4 — List of top five canonical pathways being altered in N6 (WT), N9 (Rest+/−), and N8 (Rest−/−) ESCs after prolonged culturing. (DOCX) [file pone.0043659.s013.docx]

**Table S4. List of top five canonical pathways being altered in N6 (WT), N9 (Rest+/-), and N8 (Rest-/-) ESCs after prolonged culturing**

| Genome wide expression profile reveals alterations in Canonical Pathways | | |
| --- | --- | --- |
| Name | p-value | Ratio |
| N6 (Wild-type Parental control) |  |  |
| Regulation of Actin-based Motility by Rho | 1.86E-02 | 2/92 (0.022) |
| Role of Tissue Factor in Cancer | 3.05E-02 | 2/116 (0.017) |
| B Cell Receptor Signaling | 4.95E-02 | 2/157 (0.013) |
| CXCR4 Signaling | 5.58E-02 | 2/171 (0.012) |
| Germ Cell-Sertoli Cell Junction Signaling | 5.83E-02 | 2/168 (0.012) |
| N9 (Rest+/-) |  |  |
| ILK Signaling | 2.57E-07 | 24/191 (0.126) |
| Human Embryonic Stem Cell Pluripotency | 1.55E-06 | 19/157 (0.121) |
| Hepatic Fibrosis / Hepatic Stellate Cell Activation | 1.06E-05 | 18/147 (0.122) |
| Basal Cell Carcinoma Signaling | 1.75E-05 | 12/73 (0.164) |
| Aryl Hydrocarbon Receptor Signaling | 2.44E-05 | 17/155 (0.11) |
| N8 (Rest-/-) |  |  |
| ILK Signaling | 5.45E-07 | 28/191 (0.147) |
| Role of Nanog in Mammalian Embryonic Stem Cell Pluripotency | 2.00E-06 | 20/117 (0.171) |
| Basal Cell Carcinoma Signaling | 3.70E-06 | 15/73 (0.205) |
| Caveolar-mediated Endocytosis Signaling | 7.64E-06 | 15/85 (0.176) |
| Human Embryonic Stem Cell Pluripotency | 1.08E-05 | 21/157 (0.134) |
